# Mixture-Dependent Toxicity of E-Cigarette Constituents: Cytotoxicity, Inflammation, and DNA-Repair-Associated Protein Responses in Bronchial Epithelial Cells

**DOI:** 10.3390/toxics14100910

**Published:** 2026-10-15

**Authors:** Ehsanul Kabir, Joshua Furst, Thivanka Muthumalage

**Affiliations:** School of Health Sciences, Purdue University, West Lafayette, IN 47907, USA

**Keywords:** ENDS, electronic cigarette, flavoring chemicals, WS23, nickel oxide, mixture toxicity, BEAS-2B, IL-6, IL-8, NEIL2, PNKP

## Abstract

Electronic cigarette (e-cigarette) aerosols contain complex mixtures of flavoring chemicals, synthetic cooling agents, and metals derived from device components. The biological effects of these constituents in combination remain incompletely characterized. We evaluated selected flavoring chemicals and nickel oxide (NiO), individually and in combination, in BEAS-2B human bronchial epithelial cells. Cells were exposed for 24 h to vanillin, WS23, ethyl vanillin, or p-anisaldehyde over an experimental concentration range of 10–1000 µM. Vanillin-WS23 and ethyl vanillin-p-anisaldehyde mixtures were evaluated at a fixed total concentration of 1000 µM over multiple component ratios. Separate experiments evaluated 100 µM ethyl vanillin, 50 µg/cm^2^ NiO, and their combination. Vanillin, WS23, ethyl vanillin, and p-anisaldehyde reduced viability-associated metabolic activity and cellular ATP in a concentration-dependent manner. The lowest concentrations associated with significant reductions in the MTT endpoint were 200 µM for vanillin, 50 µM for WS23, 300 µM for ethyl vanillin, and 200 µM for p-anisaldehyde. Chou-Talalay analysis identified synergistic interactions between vanillin and WS23 (combination index [CI] < 1) and antagonistic interactions between ethyl vanillin and p-anisaldehyde (CI > 1). Ethyl vanillin and NiO produced no statistically significant decrease in viability but increased inflammatory cytokines in conditioned medium. Both IL-6 and IL-8 levels were significantly elevated with NiO and the combined ethyl vanillin-NiO exposure. Total H2AX abundance did not differ significantly among groups at 24 h. NEIL2 abundance showed increasing trends following NiO alone (*p* = 0.0518) and combined ethyl vanillin–NiO exposure (*p* = 0.0760). PNKP increased significantly after ethyl vanillin (*p* = 0.0261) and combined ethyl vanillin-NiO (*p* = 0.0041) treatments. These findings show mixture-dependent biological responses that differed from the effects observed with individual constituents. Flavoring chemical mixtures produced either synergistic or antagonistic effects, and ethyl vanillin-NiO exposure produced inflammatory and DNA-repair-associated protein responses under conditions that did not significantly reduce cell viability. By demonstrating non-additive flavoring chemical interactions together with inflammatory and DNA-repair-associated protein responses to chemical-metal exposure, this study evaluated e-cigarette toxicity beyond single-constituent hazard characterization. The NEIL2 and PNKP findings support altered base-excision repair (BER) pathways but do not establish oxidative DNA lesions or increased DNA repair activity.

## 1. Introduction

Electronic cigarettes (e-cigarettes), also termed electronic nicotine delivery systems, generate aerosols from liquids that commonly contain propylene glycol, vegetable glycerin, nicotine, and flavoring chemicals. Aerosol composition also reflects device materials and operating conditions and can include carbonyl compounds and metal-containing particles derived from metallic device components [1,2,3,4]. Because these constituents are inhaled together, evaluation of individual chemicals alone may not predict the biological effects of the resulting mixture.

Many e-cigarette flavoring chemicals are used in foods, but safety for ingestion does not establish safety for inhalation [5]. Flavoring chemicals and flavored e-liquids have produced cytotoxicity, oxidative stress, inflammatory responses, and epithelial dysfunction in experimental models [6,7,8,9]. Flavor aldehydes can also react with propylene glycol and vegetable glycerin to form acetals with properties that differ from those of the parent aldehydes [10].

WS23 (2-isopropyl-N,2,3-trimethylbutyramide) is a synthetic cooling agent detected in e-cigarette products, including disposable products with cooling or menthol-like sensory characteristics [11,12]. WS23 aerosol exposure can alter airway epithelial responses, including mucin expression and epithelial signaling [13]. The effects of WS23 in mixtures with flavor aldehydes have received less study.

Mixture effects can be additive, synergistic, or antagonistic. Their direction and magnitude may depend on concentration, component ratio, chemical reactivity, cellular uptake, metabolism, and shared biological targets. The Chou-Talalay median-effect method provides a quantitative framework for assessing combined effects, with CI < 1 indicating synergism, CI = 1 additivity, and CI > 1 antagonism [14,15].

Metals represent an additional component of e-cigarette exposure. Nickel has been measured in e-cigarette liquids and aerosols, and metallic device components can contribute nickel-containing particles to e-cigarette aerosols [3,16,17]. Metal exposure from e-cigarette components has been associated with oxidative, inflammatory, cytotoxic, and genotoxic endpoints [4,18]. The cellular response to simultaneous exposure to a flavoring chemical and a nickel-containing particle is less well characterized.

Airway epithelial cells contribute to pulmonary innate immune signaling and release cytokines after toxicant exposure [19,20]. IL-6 and IL-8 are commonly measured indicators of epithelial inflammatory responses [21,22]. Inflammatory signaling can occur without significant cell death; therefore, cell viability and secreted cytokine levels provide complementary information about cellular injury.

Oxidative and inflammatory stress can also affect DNA-damage and DNA-repair pathways. Phospho-histone-H2AX at Ser 139 is a marker of DNA double-strand-break-associated signaling [23]. Total histone is known to decrease during destabilization of total histones under significant oxidative stress [24]. NEIL2 is a DNA glycosylase/AP lyase involved in repair of oxidized bases, and NEIL2-initiated repair can require polynucleotide kinase 3′-phosphatase (PNKP) to process 3′-phosphate termini [25]. NEIL2 and PNKP also contribute to maintenance of mitochondrial genome integrity [26]. Changes in abundance of these proteins, however, do not by themselves establish DNA lesion burden or repair activity.

Together, these observations highlight two complementary challenges in evaluating electronic nicotine delivery systems (ENDS) constituent safety. First, the interactions among flavoring constituents may alter the cytotoxic responses relative to those predicted from individual compounds. Second, co-exposure to chemically distinct constituents such as flavoring chemicals and metals may elicit biological responses that are not apparent from acute cytotoxicity measurements alone. Evaluating both types of mixture effects may therefore provide a more comprehensive assessment of ENDS relevant exposures than single constituents.

The present study was designed to evaluate beyond single-constituent testing by determining whether selected e-cigarette-relevant binary chemical constituents produce distinct non-additive interaction patterns and whether flavoring chemical-metal exposure produces biological responses not recapitulated by acute viability assessment alone. We determined concentration-response relationships for vanillin, WS23, ethyl vanillin, and p-anisaldehyde; evaluated vanillin-WS23 and ethyl vanillin-p-anisaldehyde mixtures using combination-index analysis; and measured viability, IL-6, IL-8, H2AX, NEIL2, and PNKP after ethyl vanillin, NiO, or combined exposure. This experimental design integrated mixture-interaction analysis with inflammatory and DNA-repair-associated protein endpoints in human bronchial epithelial cells. We hypothesized that mixture responses would differ from those predicted from individual constituents. Specifically, we tested whether flavoring-chemical mixtures exhibit distinct non-additive interaction patterns and whether metallic compounds, ethyl vanillin-NiO, co-exposure alters inflammatory and DNA-repair-associated responses at concentrations that produced limited acute cytotoxicity.

## 2. Materials and Methods

### 2.1. Cell Culture and Treatment

Human bronchial epithelial BEAS-2B cell (ATCC, Manassas, VA, USA, Catalogue No. CRL-3588, Lot No. 70060497) were cultured in Gibco Dulbecco’s Modified Eagle Medium/F-12 (DMEM/F-12) containing 5% fetal bovine serum (FBS), 1% glutamine, 1% antibiotic-antimycotic solution, and 15 mM HEPES. Cells were cultured at 37 °C in a humidified atmosphere containing 5% CO_2_. At approximately 80–85% confluence, cells were serum-deprived for 16–18 h in DMEM/F-12 containing 1% FBS. Treatments were initiated at approximately 90–95% confluence. Conditioned medium and cell pellets were collected 24 h after exposure and stored at −80 °C until analysis.

Cell-line authentication and mycoplasma testing: BEAS-2B cell line was authenticated through short tandem repeat (STR) profiling by ATCC, which confirmed identity and purity. We validated mycoplasma free cultures (InvivoGen, San Diego, CA, USA, Cat No. rep-mys-50).

### 2.2. Flavoring Chemicals, NiO, and Exposure Conditions

Flavoring-chemical stock solutions were prepared at 100 mM stock concentration in 100% ethanol. The final vehicle concentration in culture medium was ≤1% ethanol and we used vehicle-matched 1% ethanol control for the experiments.

Vanillin (Thermo Scientific, Waltham, MA, USA, Cat. No. A11169.22, Lot No. P30I039), WS23 (TCI, Tokyo, Japan, Cat. No. I0729, Lot No. BUWKA-FF), ethyl vanillin (Sigma-Aldrich, St. Louis, MO, USA, Cat. No. PHR1268, Lot No. LRAD8136), and p-anisaldehyde (TCI, Tokyo, Japan, Cat. No. A0480, Lot No. TUBBF-BZ) were evaluated individually and in binary mixtures. Individual concentration-response experiments evaluated a broad experimental concentration range of 10–1000 µM for the MTT assay to characterize concentration-dependent responses rather than focusing only on high-dose effects. CellTiter-Glo experiments used concentrations from 25 to 1000 µM. Cells were exposed for 24 h.

Vanillin-WS23 and ethyl vanillin-p-anisaldehyde mixtures were prepared at a fixed total concentration of 1000 µM. Vanillin-WS23 and ethyl vanillin-p-anisaldehyde mixtures were evaluated at multiple component ratios. The total final nominal concentration was held constant at 1000 µM while the relative proportions of the two constituents were varied to evaluate mixture interactions across component ratios at a fixed total concentration. Individual treatment concentration for all chemicals were 10, 25, 50, 100, 200, 300, 400, 500, 600, 700, 800, 900, and 1000 µM for MTT assay, and 25, 50, 100, 250, 500, 750, 1000 µM for CellTiter-Glo assay. Ratio series for the binary mixture was 1:1, 1:3, 1:4, 1:9, 1:19, 1:24, 1:49, 3:1, 4:1, 9:1, 19:1, 24:1, and 49:1 for both experiments using their individual doses in MTT assay, and CellTiter-Glo included only 1:1, 1:3, 1:9, 3:1 and 9:1 mixed ratios.

For flavoring chemical-metal experiments, passage-14 BEAS-2B cells were exposed for 24 h to 100 µM ethyl vanillin, 50 µg/cm^2^ NiO, or 100 µM ethyl vanillin plus 50 µg/cm^2^ NiO. Based on prior dose-response studies of ethyl vanillin and NiO, 100 µM ethyl vanillin and 50 µg/cm^2^ NiO were selected for the combination experiment because these concentrations did not significantly reduce the MTT endpoint individually, allowing evaluation of inflammatory and DNA-repair-associated responses without substantial loss of viability. The NiO dose was expressed as nominal administered mass per unit culture-well growth area; thus, 50 µg/cm^2^ represents 50 µg of NiO added per cm^2^ of culture-well growth area and does not represent a dissolved concentration or a measured deposited dose on the cell monolayer. Nickel oxide (NiO; Thermo Scientific, Waltham, MA, USA, Product No. 012359, Lot No. Y25K026, purity 99%) had a lot-specific Certificate of Analysis reporting a screen analysis of −400 mesh, with 100% passing. This screen specification does not represent a measured particle-size distribution. NiO was prepared as a 1 mg/mL suspension. Pluronic F-68 (0.05%) was included to improve particle dispersion/stability and limit agglomeration during suspension preparation. The suspension was sonicated for 10 min to reduce clumping, vortexed, and used immediately after preparation.

Component concentrations, mixture ratios, fraction affected, and combination-index values are provided in Supplementary Table S1. Experimental replication and statistical details are summarized in Supplementary Table S2.

### 2.3. MTT Assay

BEAS-2B cells were seeded in 96-well plates at approximately 10,000 cells per well in 200 µL of medium containing 5% FBS. Following serum deprivation in medium containing 1% FBS, cells were exposed to vanillin, WS23, ethyl vanillin, p-anisaldehyde, or the indicated binary mixtures. The total fixed concentration of each binary mixture was 1000 µM. After 24 h, treatment medium was replaced with fresh medium, 10 µL MTT solution was added to each well, and plates were incubated for 4 h at 37 °C. SDS-HCl solution (100 µL) was then added to each well, followed by a second 4-h incubation at 37 °C. After mixing, absorbance was measured at 570 nm. The MTT response was calculated as (absorbance of treated cells/absorbance of untreated control) × 100. MTT assays were repeated three times, with two technical replicate wells per treatment condition in each repeat. MTT reduction was interpreted as viability-associated metabolic activity rather than as a direct measure of cell number [27].

### 2.4. CellTiter-Glo ATP Assay

Passage-10 BEAS-2B cells were seeded at 15,000 cells per well in white opaque 96-well plates. At 80–85% confluence, cells were serum-deprived for 16 h in medium containing 1% FBS and then exposed to vanillin, WS23, ethyl vanillin, p-anisaldehyde, or their mixtures. Individual concentrations ranged from 25 to 1000 µM. Cellular ATP was measured 24 h after treatment using CellTiter-Glo 2.0 (Promega, Madison, WI, USA, Cat. No. G9241). ATP standards were 0, 1 nM, 10 nM, 100 nM, 1 µM, 10 µM, and 100 µM. CellTiter-Glo reagent (100 µL) was added to each well, plates were mixed for 2 min and incubated for 10 min to stabilize luminescence, and luminescence was recorded. ATP concentrations were calculated from the standard curve. Luminescence was measured using SpectraMax Id3, Molecular Devices (San Jose, CA, USA).

### 2.5. Trypan Blue Exclusion Assay

At cell collection, 10 µL of cell suspension was mixed with 10 µL trypan blue. Ten microliters of the mixture were loaded into a hemocytometer, and trypan-blue-excluding and trypan-blue-positive cells were counted by microscopy. Percent viability was calculated as the number of trypan-blue-excluding cells divided by the total number of cells counted × 100.

### 2.6. Combination-Index Analysis

Chemical interactions were evaluated using the Chou-Talalay median-effect method in CompuSyn (ComboSyn Inc., version 1.0, Paramus, NJ, USA) [14,15]. MTT-derived dose-effect data for individual chemicals and binary mixtures were entered into CompuSyn to calculate fraction affected (Fa) and CI. CI < 1 indicated synergism, CI = 1 additivity, and CI > 1 antagonism.

All the CI calculations were done based on experiment-level data. We entered all the mixed ratios from MTT assay data as non-constant combo in CompuSyn and used the respective effect (Fa) and CI values to generate Fa-CI curve (Table S1).

### 2.7. IL-6 and IL-8 ELISA

Conditioned medium was collected 24 h after treatment. Secreted IL-6 and IL-8 were quantified using DuoSet ELISA kits (R&D Systems; Minneapolis, MN, USA, IL-6, Cat. No. DY206; IL-8, Cat. No. DY208) according to the manufacturer’s protocol. Cytokine concentrations were reported as pg/mL conditioned medium.

### 2.8. Immunoblotting

Cells were lysed in RIPA buffer (Thermo Scientific, Waltham, MA, USA, Cat No. J63306.AP) containing protease inhibitor (Thermo Scientific, Waltham, MA, USA, Cat No. 87785), and total protein was quantified using a BCA assay kit (Thermo Fisher Scientific, Waltham, MA, USA, Cat. No. 23225). Twenty micrograms of protein per sample was loaded onto 4–10% polyacrylamide gels (10 wells, 1.5 mm) and separated by electrophoresis at 120 V. Proteins were transferred to polyvinylidene difluoride membranes using a Bio-Rad Trans-Blot Turbo system. Transfer was confirmed by Ponceau S staining (Thermo Fisher Scientific, Waltham, MA, USA, Cat. No. A40000279).

Membranes were blocked for 1 h at room temperature with gentle shaking in 5% nonfat milk prepared in 1× Tris-buffered saline containing 0.1% Tween-20 (TBST), followed by three 10-min washes in TBST. Membranes were incubated overnight at 4 °C with antibodies against H2AX (R&D Systems, Minneapolis, MN, USA, Cat. No. MAB3406), NEIL2 (Novus Biologicals, Centennial, CO, USA, Cat. No. NBP1-98526), or PNKP (Thermo Fisher Scientific, Waltham, MA, USA, Cat. No. MA5-55330), each at 1:1000 in 5% milk/TBST.

After three TBST washes, membranes were incubated with HRP-conjugated goat anti-rabbit IgG (Abcam, Cambridge, UK Cat. No. AB6721; 1:5000) for NEIL2 and PNKP or HRP-conjugated goat anti-mouse IgG (Abcam, Cambridge, UK Cat. No. AB97040; 1:5000) for H2AX. Membranes were washed three times and developed using SuperSignal West Femto chemiluminescent substrate (Thermo Fisher Scientific, Waltham, MA, USA, Cat. No. 34094). Images were acquired with a Bio-Rad ChemiDoc XRS+ system.

Membranes were stripped with stripping buffer (Thermo Scientific, Waltham, MA, USA, Cat No. 46430), blocked again for 1 h in 5% milk/TBST, washed three times, and reprobed overnight with Lamin B1 antibody (Abcam, Cambridge, UK, Cat. No. AB16048-1001). Band density was quantified using Bio-Rad Image Lab software (Version 6.1, Bio-Rad Laboratories). H2AX, NEIL2, and PNKP were normalized to Lamin B1. Theoretical molecular weights—H2AX, 15 kDa; NEIL2, 36.8 kDa; PNKP, 57.1 kDa; and Lamin B1, 66.4 kDa. Full-length, uncropped blots are provided in the Supplementary Materials.

### 2.9. Statistical Analysis

*Statistical analyses were performed using GraphPad Prism version 10.5.0. Data are presented as mean ± SEM. Comparisons among treatment groups were performed using one-way analysis of variance followed by Tukey’s multiple-comparisons test. Adjusted *p* < 0.05 was considered statistically significant. Figure 1 contains *n* = 2–4 observations depending on the panel, Figure 2 contains *n* = 3–12, Figure 3 contains *n* = 3, and immunoblot analyses contain *n* = 2–3 (Figure 4). In all cases, *n* number denotes independent biological replicates. For Figure 1, Figure 2 and Figure 3, we used 2 technical replicates too, and immunoblotting used all biological replicates where *n* number was 2–3 to fit randomly selected samples in 10 well gels. We did not exclude any data points from experimental groups during analysis. 

## 3. Results

### 3.1. Vanillin and WS23 Reduce Metabolic Activity and Produce Synergistic Effects in Mixture

Vanillin reduced the MTT endpoint significantly at 200, 400, 600, 700, 800, 900, and 1000 µM relative to untreated control (Figure 1A). WS23 significantly reduced the MTT endpoint at 50, 200, 300, 400, 500, 600, 700, 800, 900, and 1000 µM (Figure 1B). Thus, the lowest tested concentrations associated with significant effects were 200 µM for vanillin and 50 µM for WS23. 

Vanillin-WS23 mixtures were evaluated at a fixed total concentration of 1000 µM over multiple component ratios. All tested mixtures significantly reduced the MTT endpoint relative to control, and mixtures containing higher proportions of vanillin produced reductions greater than 50% in MTT-based viability-associated metabolic activity in some conditions (Figure 1C). CI values were <1 for all evaluated mixtures, consistent with synergistic interactions under the experimental conditions (Figure 1D). CellTiter-Glo measurements showed concentration-dependent ATP depletion after individual exposures, and mixtures containing higher proportions of vanillin produced greater ATP depletion. The 1000 µM vanillin condition and the 9:1 vanillin:WS23 mixture were significantly decreased from control (Figure 1E).

### 3.2. Ethyl Vanillin and P-Anisaldehyde Produce Antagonistic Effects in Mixture

Passage-20 BEAS-2B cells were exposed for 24 h to ethyl vanillin, p-anisaldehyde, or binary mixtures at a fixed total concentration of 1000 µM. Ethyl vanillin significantly reduced the MTT endpoint at concentrations from 300 to 1000 µM (Figure 2A). p-Anisaldehyde significantly reduced the MTT endpoint at concentrations from 200 to 1000 µM (Figure 2B).

Binary mixtures reduced the MTT endpoint, but mixtures containing higher proportions of p-anisaldehyde were generally not significantly different from control, whereas mixtures containing higher proportions of ethyl vanillin showed significant effects (Figure 2C). CI values were >1 for all evaluated ethyl vanillin-p-anisaldehyde mixtures, consistent with antagonistic interactions under the experimental conditions (Figure 2D). CellTiter-Glo measurements showed concentration-dependent ATP depletion after ethyl vanillin exposure, and mixtures containing higher proportions of ethyl vanillin produced greater ATP depletion (Figure 2E).

We established that flavoring chemical mixtures could produce distinct non additive interaction patterns, we next examined whether co-exposure to chemically dissimilar ENDS constituents could elicit biological responses under conditions without significant acute toxicity. Ethyl vanillin and NiO were therefore evaluated individually and in combination using toxicity and inflammatory end points.

### 3.3. Ethyl Vanillin and NiO Increase Inflammatory Cytokines Without a Significant Decrease in Viability

Passage-14 BEAS-2B cells were exposed for 24 h to 100 µM ethyl vanillin, 50 µg/cm^2^ NiO, or their combination. Trypan blue exclusion showed no statistically significant difference in percent viability among treatment groups and control (Figure 3A).

IL-6 concentrations were significantly increased after NiO, and combined ethyl vanillin-NiO exposure relative to untreated control (Figure 3B). IL-6 was also significantly higher after combined exposure than after NiO alone. IL-8 concentrations were significantly increased after NiO and combined exposure relative to control and ethyl vanillin alone (Figure 3C). These findings show that inflammatory responses occurred at exposure conditions that did not produce a statistically significant decrease in cell viability.

### 3.4. Ethyl Vanillin and NiO Exposures Are Associated with Differential NEIL2 and PNKP Protein Abundance Without Changes in Total H2AX at 24 h

Total H2AX abundance did not differ significantly among control, ethyl vanillin, NiO, and combined ethyl vanillin-NiO groups at 24 h (Figure 4A).

NEIL2 abundance showed a nonsignificant increasing trend after NiO alone relative to control (Tukey-adjusted *p* = 0.0518). For ethyl vanillin + NiO versus control, NEIL2 abundance was increased with a nonsignificant trend (Tukey-adjusted *p* = 0.0760) (Figure 4B).

PNKP abundance increased significantly after ethyl vanillin (Tukey-adjusted *p* = 0.0261) and combined ethyl vanillin-NiO exposure (Tukey-adjusted *p* = 0.0041) relative to control. Combined exposure also produced significantly greater PNKP abundance than NiO alone (Tukey-adjusted *p* = 0.0174) (Figure 4C).

## 4. Discussion

This study demonstrated that selected-e-cigarette-relevant mixtures produced opposite interaction phenotypes in the same bronchial epithelial model: vanillin-WS23 mixtures yielded CI values consistent with synergism, whereas ethyl vanillin-p-anisaldehyde mixtures yielded CI values consistent with antagonism. A second contribution is that ethyl vanillin-NiO exposure elicited inflammatory cytokine responses under conditions without a statistically significant loss of cell viability, demonstrating a biological response that was not captured by the acute viability endpoint. Immunoblot data identified DNA-BER-associated responses. Although NEIL2 abundance showed an increasing trend in NiO-containing treatment conditions and PNKP abundance increased in ethyl vanillin-containing conditions, total H2AX abundance did not differ significantly among groups at 24 h, suggesting that the induced oxidative stress was not associated with detectable histone degradation. These findings suggest that mixture composition influenced NEIL2-associated DNA-repair responses involving proteins that participate in base excision repair due to oxidative stress.

The study was designed to first establish concentration-response relationships for individual constituents and then evaluate selected mixtures to determine whether their combined effects were additive, synergistic, or antagonistic. The two flavoring-chemical mixture models produced distinct interaction patterns: vanillin-WS23 showed synergistic interactions, whereas ethyl vanillin-p-anisaldehyde showed antagonistic interactions under the experimental conditions. We subsequently evaluated ethyl vanillin-NiO co-exposure using concentrations selected from prior dose-response studies to examine inflammatory and DNA-repair-associated responses under conditions without substantial loss of viability. Together, this design allowed evaluation of mixture-dependent biological responses that may not be predicted from single-constituent testing alone.

WS23 affected the MTT endpoint at a lower concentration than the other flavoring chemicals tested. WS23 is present in marketed e-cigarette products and has been shown to alter airway epithelial responses [11,12,13]. Vanillin has also produced cytotoxic effects in bronchial epithelial models [9]. In the present study, CI values < 1 across the evaluated vanillin-WS23 mixtures indicate that the combined effects exceeded those predicted from the individual dose-effect relationships. Synergistic effects have also been reported for other aldehyde mixtures in BEAS-2B cells [28].

The mechanism underlying vanillin-WS23 synergy was not examined. Potential explanations include changes in cellular uptake, membrane properties, mitochondrial function, or convergence on common stress-response pathways. Flavor aldehydes can react with e-liquid solvents to form acetals [10], but the present study did not evaluate chemical reaction products. The current data therefore establish the interaction phenotype but do not identify its chemical or molecular basis.

Relatively high concentrations of flavoring chemicals have been reported in commercial e-liquids. Vanillin concentrations up to 31 mg/mL have been measured in refill fluids [29], and a survey of 277 refill fluids found that 37% contained total flavor chemical concentrations > 10 mg/mL, with ethyl vanillin exceeding 10 mg/mL in some products [30]. These product measurements provide context for the concentration range evaluated here but are not directly equivalent to nominal concentrations used in submerged cell culture or to concentrations delivered to the human airway. Accordingly, the 1000 µM total concentration used in the vanillin-WS23 mixture experiment should be interpreted as an experimental condition used to evaluate mixture interactions at a fixed total concentration rather than as a directly equivalent human airway exposure. Ethyl vanillin-p-anisaldehyde mixtures produced CI values > 1, indicating antagonism. In combination-index analysis, antagonism means that the measured combined effect is lower than that predicted from the individual dose-effect relationships; it does not indicate absence of toxicity [14,15]. Antagonistic interactions have been reported for other vaping ingredients, including limonene and triethyl citrate [31]. Additional dose ranges and mixture ratios would be required to determine whether the interaction observed here is maintained across a broader effect range.

The ethyl vanillin-NiO experiment examined a different component of mixture toxicity. Nickel is relevant to e-cigarette exposure because metallic device components can contribute nickel-containing particles to e-cigarette aerosols [3,16,17]. A previous study also showed that ethyl maltol enhanced copper-mediated cytotoxicity in lung epithelial cells, demonstrating that flavoring compounds can modify cellular responses to metals [32]. In the present study, 100 µM ethyl vanillin and 50 µg/cm^2^ NiO did not significantly reduce cell viability, but they altered cytokine release.

IL-6 increased after ethyl vanillin, NiO, and combined exposure, and the combination produced higher IL-6 than NiO alone. IL-8 increased after NiO and combined exposure. Airway epithelial cells are active participants in pulmonary inflammatory signaling [19,20]. The cytokine results therefore indicate a biological response that was not reflected by the cell viability endpoint.

The DNA-repair-associated protein pattern identifies a hypothesis for mechanistic follow-up. NEIL2 abundance showed a nonsignificant increasing trend after NiO alone, whereas PNKP increased after ethyl vanillin and combined exposure. NEIL2 is a DNA glycosylase/AP lyase involved in processing selected oxidized bases, and NEIL2-initiated repair of 5-hydroxyuracil has been shown to require polynucleotide kinase activity to generate repair-compatible 3′-OH termini [25,33]. NEIL2 and PNKP also contribute to mitochondrial genome maintenance [26]. The protein abundance changes observed here therefore support altered base-excision-repair pathways, but protein abundance alone does not establish oxidative DNA lesions, pathway activation, or increased DNA repair activity.

Total H2AX abundance did not differ significantly among groups at 24 h. The consistency between the elevated NEIL2 trend, increased PNKP abundance, and the absence of significant H2AX destabilization is biologically interesting and raises the hypothesis that these exposures may be associated with altered base-damage-response pathways without producing a prominent persistent H2AX-associated double-strand-break signal at the measured time point. This interpretation does not exclude oxidative base lesions, single-strand intermediates, transient DNA damage, or damage repaired before collection [23], and it does not establish activation of base excision repair. Direct measurements of DNA lesions and repair activity are required to test this hypothesis.

Inflammation provides a plausible context for further mechanistic study because inflammatory processes can generate reactive oxygen and nitrogen species capable of damaging DNA [34]. NEIL2 expression has been altered in models of chemically induced oxidative stress [35], and ATM-dependent stabilization of PNKP has been linked to cellular resistance to oxidative stress [36]. Direct measurement of reactive oxygen species, oxidized DNA bases, and DNA strand-break intermediates would be required to connect the inflammatory response observed here with the changes in NEIL2 and PNKP.

The NEIL2 abundance directionality provides a testable hypothesis for mechanistic follow-up. A time-course experiment could determine whether cytokine induction precedes changes in NEIL2 and PNKP. Oxidative DNA lesions could be measured by 8-oxo-2′-deoxyguanosine analysis or lesion-sensitive comet assays [37,38]. NEIL2 loss-of-function experiments would determine whether NEIL2 affects lesion burden, inflammatory signaling, or cell survival after NiO and ethyl vanillin-NiO exposure. Such experiments would distinguish an exposure-associated change in NEIL2 abundance from a functional role in the cellular response.

This study has limitations. BEAS-2B cells are an immortalized bronchial epithelial model and do not reproduce the cellular diversity or differentiation state of the human airway. Exposures were performed in submerged culture using concentrations rather than aerosol exposure at an air-liquid interface. The study evaluated a single 24-h exposure period and a limited set of binary mixtures. Replicate numbers were limited for selected immunoblot analyses. Direct measures of reactive oxygen species, oxidative DNA lesions, and DNA repair activity were not included. The particle-size distribution and agglomeration state of the NiO suspension under the cell-culture exposure conditions were not directly characterized. The −400-mesh screen specification reported by the manufacturer does not constitute a measured particle-size distribution. The particle size and agglomeration could influence particle-cell interactions and biological responses; therefore, direct comparison with the smaller metal-containing particles reported in e-cigarette aerosols is limited. Future studies using size-characterized NiO particles, including smaller particles, and aerosol-based exposure approaches such as air-liquid interface exposure are needed to evaluate particle-size-dependent responses. These limitations restrict mechanistic interpretation and extrapolation to human inhalation exposure.

The results nevertheless establish that the biological effects of the tested mixtures depended on mixture composition. Vanillin-WS23 and ethyl vanillin-p-anisaldehyde produced opposite interaction patterns, and ethyl vanillin-NiO exposure produced inflammatory and DNA-repair-associated responses without significant acute loss of viability. These observations support evaluation of e-cigarette constituents as mixtures rather than assuming that combined effects can be predicted from individual-compound toxicity.

## 5. Conclusions

Vanillin, WS23, ethyl vanillin, and p-anisaldehyde produced concentration-dependent effects on viability-associated metabolic activity and cellular ATP in BEAS-2B cells. Chou-Talalay analysis yielded CI values < 1 for the tested vanillin-WS23 mixtures and CI values > 1 for the tested ethyl vanillin-p-anisaldehyde mixtures, consistent with synergistic and antagonistic interactions, respectively, under the experimental conditions. Thus, the effects of the tested flavoring chemical mixtures were dependent on mixture composition and were not described by a single assumption of additivity.

## Figures and Tables

**Figure 1:**
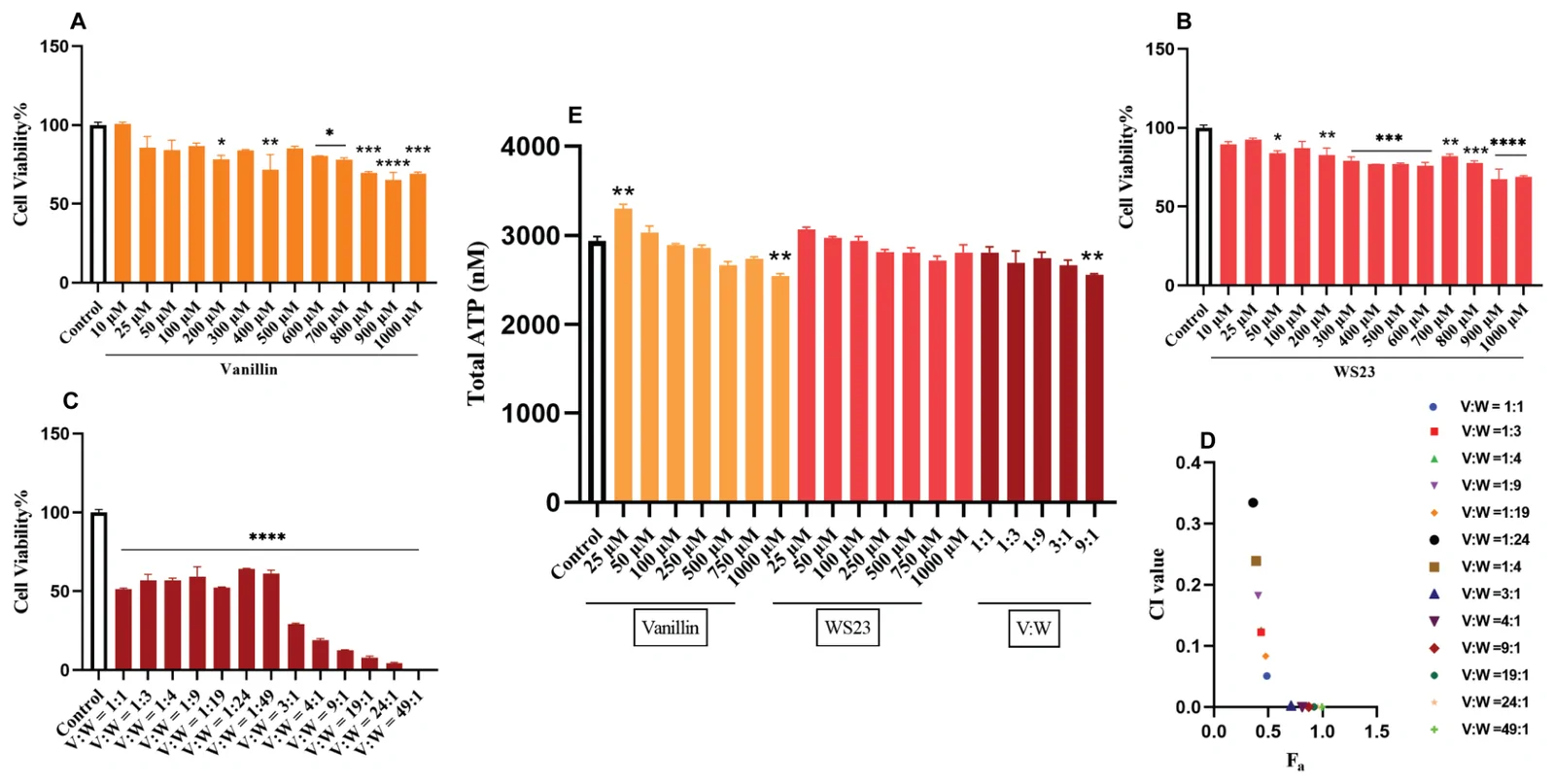
Vanillin and WS23 produce synergistic effects on viability-associated metabolic activity and cellular ATP in BEAS-2B cells. Passage-10 BEAS-2B Cells were exposed for 24 h to vanillin, WS23, or vanillin-WS23 mixtures at a fixed total concentration of 1000 µM. (**A**) MTT response to 10–1000 µM vanillin. (**B**) MTT response to 10–1000 µM WS23. (**C**) MTT response to vanillin:WS23 mixtures at the tested component ratios. (**D**) Fraction affected (Fa)-combination index (CI) plot. CI < 1 indicates synergism, CI = 1 additivity, and CI > 1 antagonism. (**E**) Cellular ATP measured by CellTiter-Glo. Data are mean ± SEM (*n* = 2–4). Statistical significance was determined by one-way ANOVA with Tukey’s multiple-comparisons test. * *p* < 0.05, ** *p* < 0.01, *** *p* < 0.001, **** *p* < 0.0001 versus control. Here, *n* = 2–4 and *n* represent independent biological replicates.

**Figure 2:**
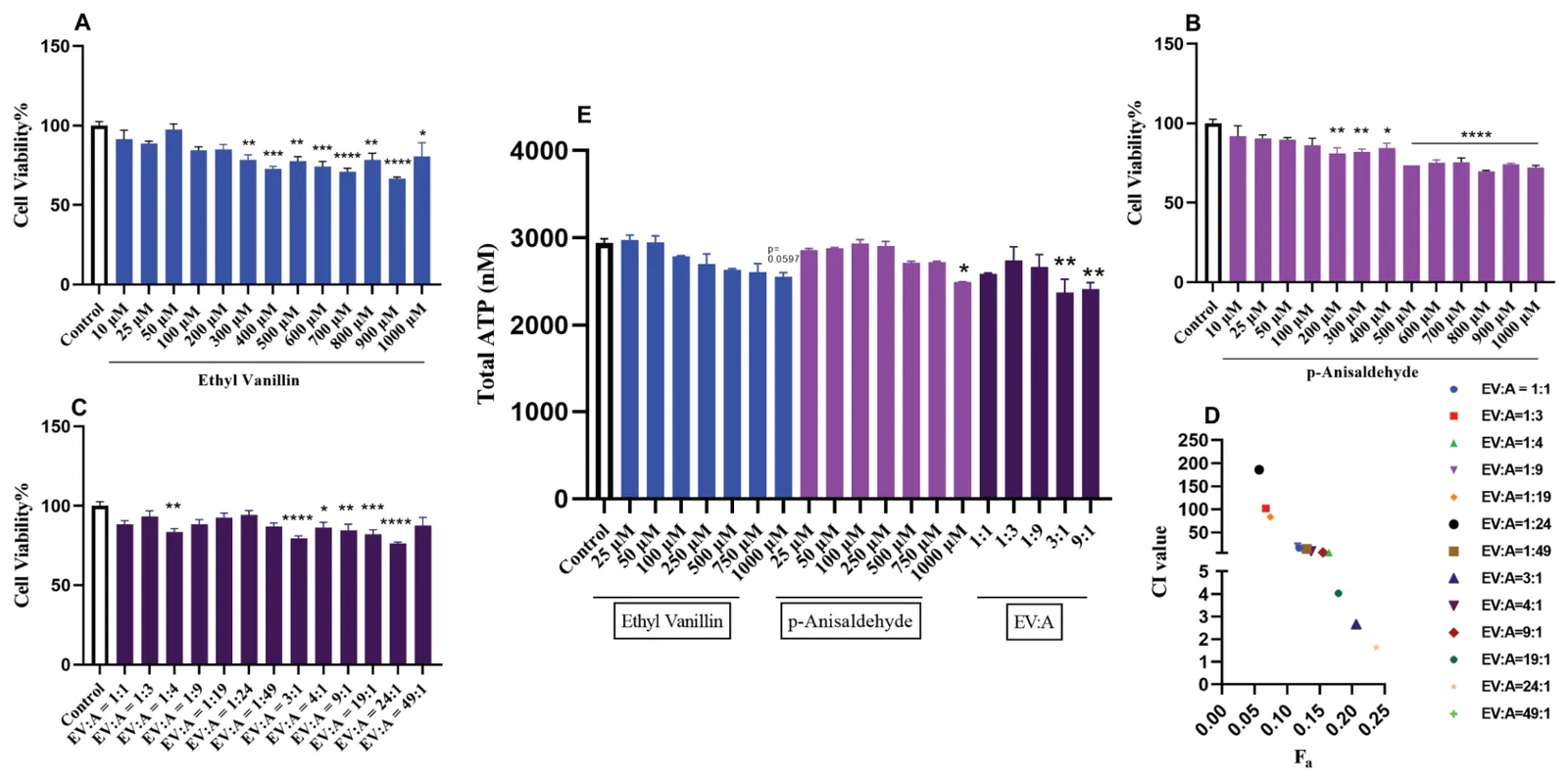
Ethyl vanillin and p-anisaldehyde produce antagonistic effects in BEAS-2B cells. Passage-20 BEAS-2B cells were exposed for 24 h to ethyl vanillin, p-anisaldehyde, or mixtures at a fixed total concentration of 1000 µM. (**A**) MTT response to 10–1000 µM ethyl vanillin. (**B**) MTT response to 10–1000 µM p-anisaldehyde. (**C**) MTT response to ethyl vanillin:p-anisaldehyde mixtures. (**D**) Fa-CI plot; CI > 1 indicates antagonism. (**E**) Cellular ATP measured by CellTiter-Glo. Data are mean ± SEM (*n* = 3–12, *n* = 2–4). Statistical significance was determined by one-way ANOVA with Tukey’s multiple-comparisons test. * *p* < 0.05, ** *p* < 0.01, *** *p* < 0.001, **** *p* < 0.0001 versus control. Here, *n* = 3–12 for MTT assay and *n* = 2–4 for CellTiter-Glo assay and n represents independent biological replicates.

**Figure 3:**
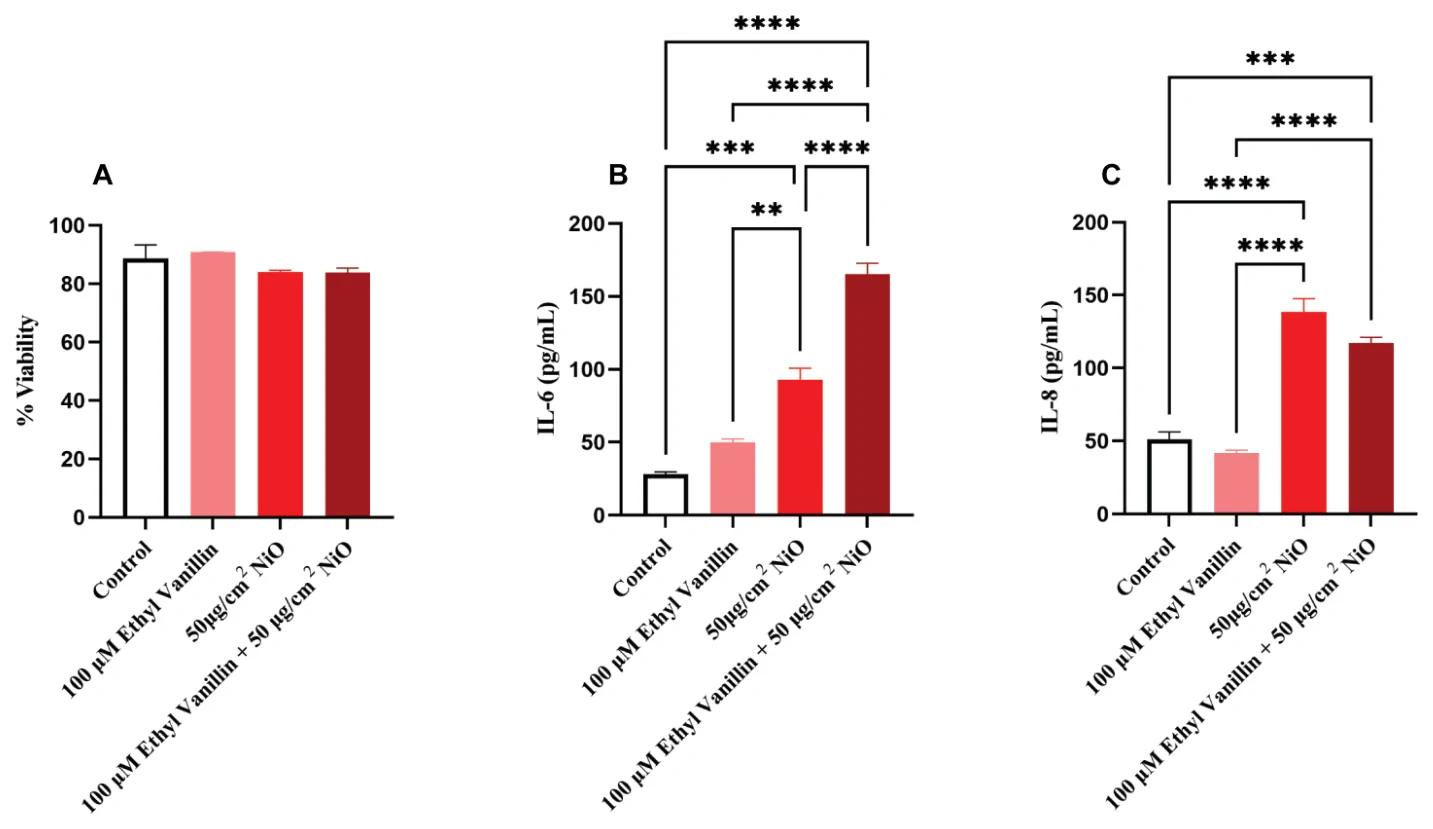
Ethyl vanillin and NiO increase inflammatory cytokines without a significant decrease in viability. Passage-14 BEAS-2B cells were exposed for 24 h to control, 100 µM ethyl vanillin, 50 µg/cm^2^ NiO, or 100 µM ethyl vanillin plus 50 µg/cm^2^ NiO. (**A**) Percent viability measured by trypan blue exclusion. (**B**) IL-6 concentration in conditioned medium. (**C**) IL-8 concentration in conditioned medium. Data are mean ± SEM (*n* = 3). Statistical significance was determined by one-way ANOVA with Tukey’s multiple-comparisons test. ** *p* < 0.01, *** *p* < 0.001, **** *p* < 0.0001 versus control.

**Figure 4:**
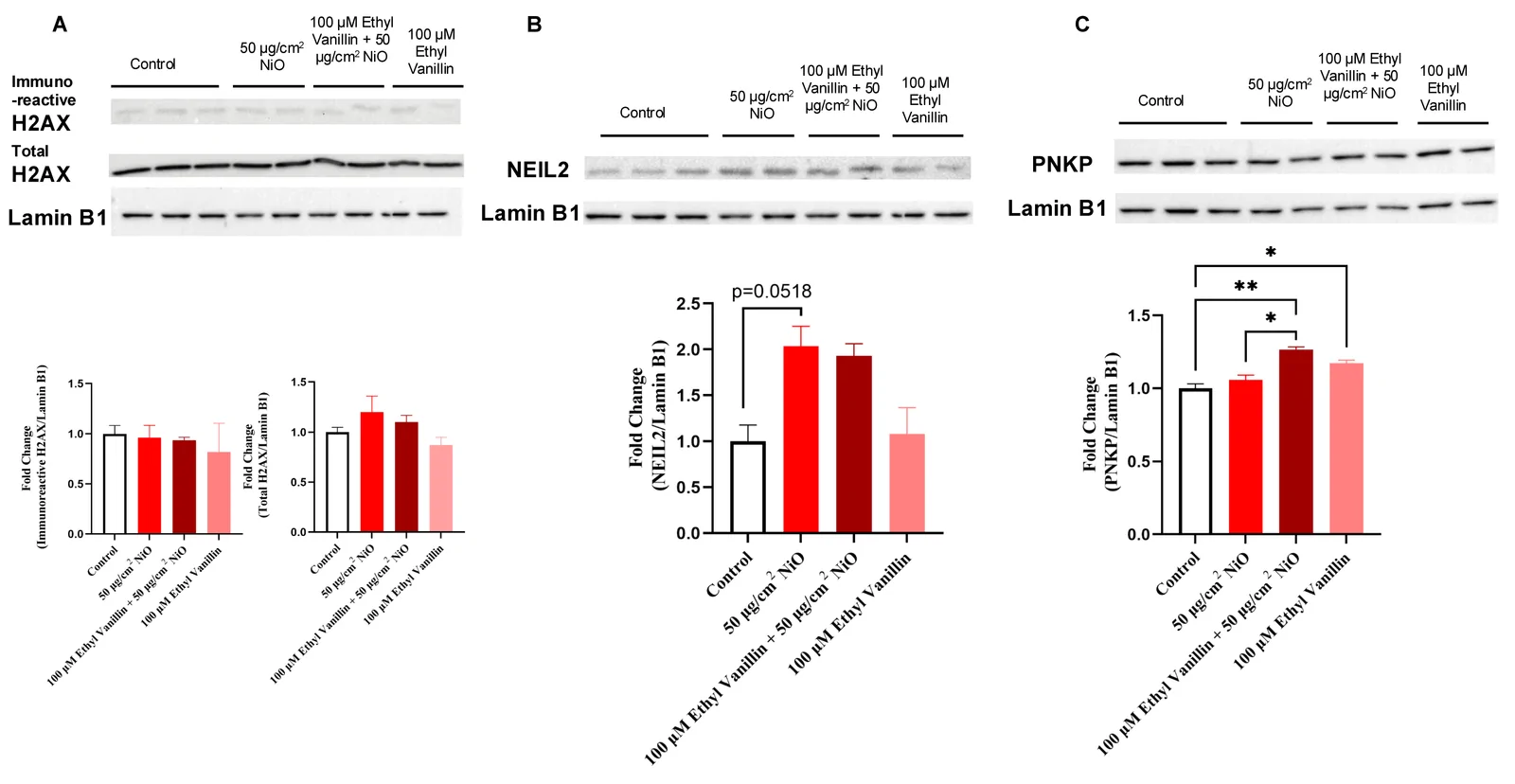
Ethyl vanillin and NiO exposures are associated with differences in abundance of DNA-repair-associated proteins without a significant difference in total H2AX abundance at 24 h. Passage-14 BEAS-2B cells were exposed for 24 h to control, 100 µM ethyl vanillin, 50 µg/cm^2^ NiO, or the combination. Cell lysates were analyzed by immunoblotting using 20 µg total protein per sample. Representative immunoblots and corresponding densitometric analyses are shown for (**A**) Immunoreactive (~25 kDa) and total H2AX (~15 kDa), (**B**) NEIL2 (~37 kDa), and (**C**) PNKP (~57 kDa). Lamin B1 (~70 kDa) was used for normalization, and densitometric values are expressed as fold change relative to control. Data are presented as mean ± SEM from independent biological replicates (*n* = 2–3). Statistical significance was determined by one-way ANOVA with Tukey’s multiple-comparisons test. * *p* < 0.05, ** *p* < 0.01, versus control.

## Data Availability

The data presented in this study are available from the corresponding author upon reasonable request. Supporting experimental and analytical information is provided in the Supplementary Materials.

## Supplementary Materials

The following supporting information can be downloaded at: https://www.mdpi.com/article/10.3390/toxics14100910/s1, Figure S1: Full-length, uncropped H2AX and Lamin B1 immunoblots with molecular-weight markers for samples from control, ethyl vanillin, and NiO treatments from Figure 4A; Figure S2: Full-length, uncropped NEIL2 and Lamin B1 immunoblots with molecular-weight markers for samples from control, ethyl vanillin, and NiO treatments from Figure 4B; Figure S3: Full-length, uncropped PNKP and Lamin B1 immunoblots with molecular-weight markers for samples from control, ethyl vanillin, and NiO treatments from Figure 4C; Table S1: Exact component concentrations, ratios, fraction affected (Fa), and combination index (CI) for all binary mixtures; Table S2: Experimental replication and statistical details for each figure panel.
