# Supplementary material for: Mixture-Dependent Toxicity of E-Cigarette Constituents: Cytotoxicity, Inflammation, and DNA-Repair-Associated Protein Responses in Bronchial Epithelial Cells
Source: Toxics. 2026 Oct 15;14(10):910. doi: 10.3390/toxics14100910 (PMC13709782; doi:10.3390/toxics14100910)
Supplement: Supplementary file 1 [file toxics-14-00910-s001.zip › toxics-4591193-supplementary.pdf]

Supplementary Materials

Mixture-Dependent Toxicity of E-Cigarette Constituents: Cytotoxicity, Inflammation, and DNA-Repair-Associated Protein Responses in Bronchial Epithelial Cells

Ehsanul Kabir, Joshua Furst and Thivanka Muthumalage *

School of Health Sciences, Purdue University, West Lafayette, IN 47907, USA; kabir4@purdue.edu (E.K.); furstj@purdue.edu (J.F.)

* Correspondence: tmuthuma@purdue.edu; Tel.: +1-(765)-496-5313; +1-(765)-494-5065

Supplementary Figure S1.

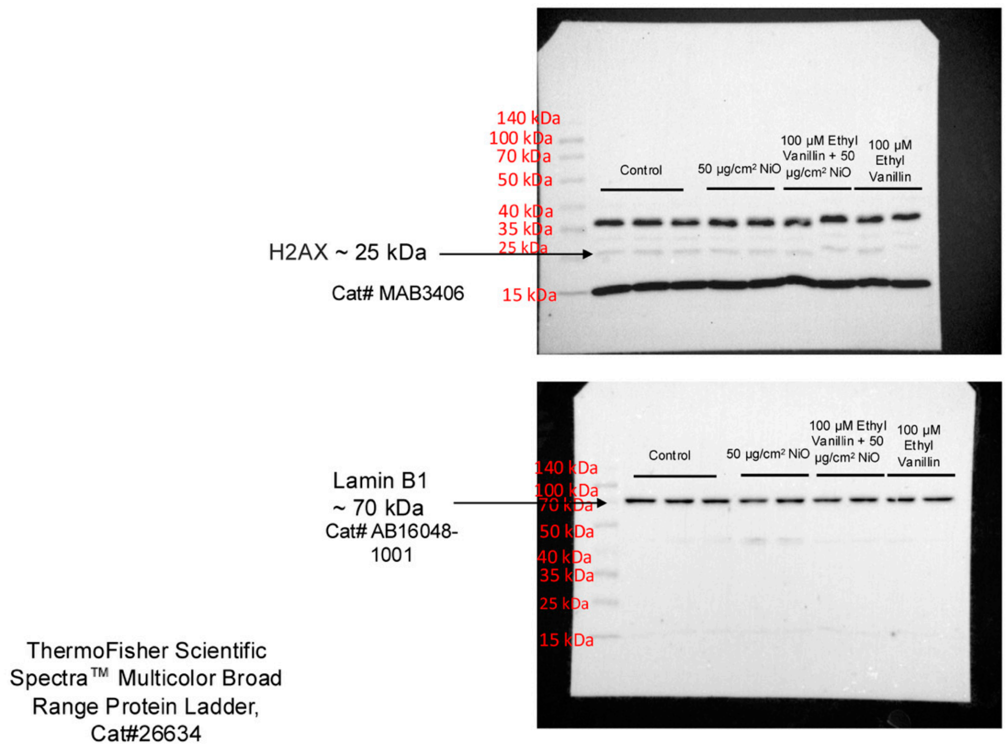

Supplementary Figure S1. Full-length, uncropped H2AX and Lamin B1 immunoblots with molecular-weight markers for samples from control, ethyl vanillin, and NiO treatments from Fig 4A.

Supplementary Figure S2.

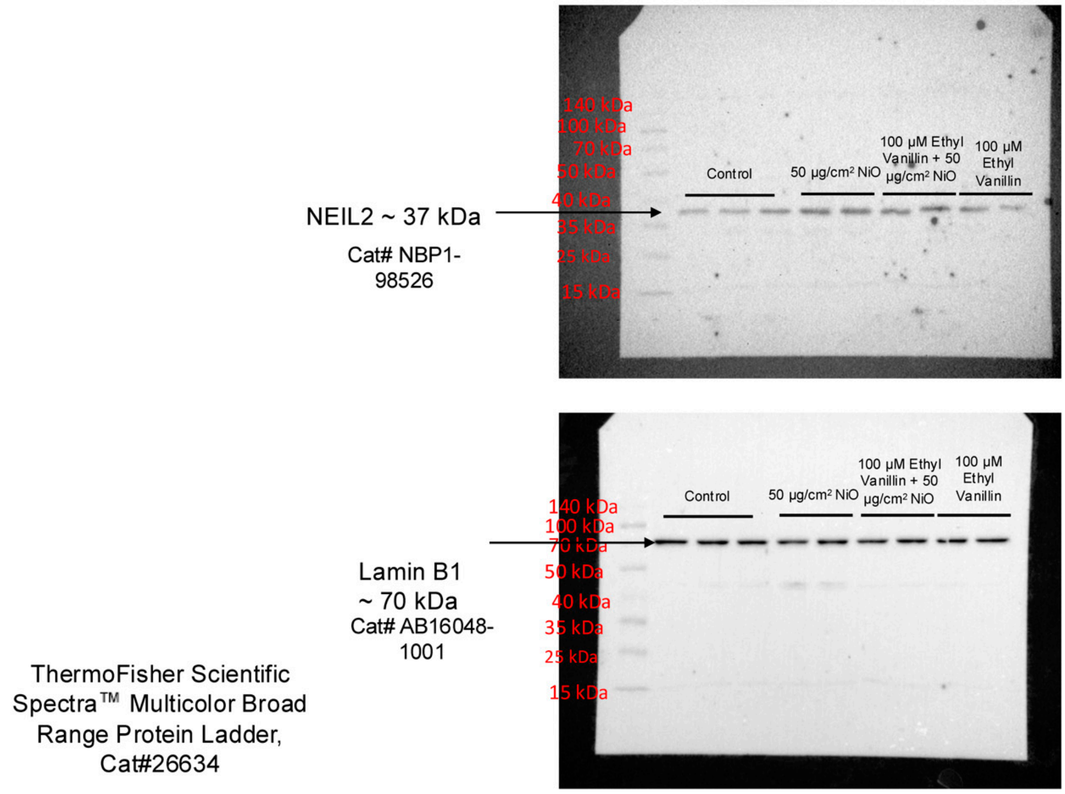

Supplementary Figure S2. Full-length, uncropped NEIL2 and Lamin B1 immunoblots with molecular-weight markers for samples from control, ethyl vanillin, and NiO treatments from Fig 4B.

Supplementary Figure S3.

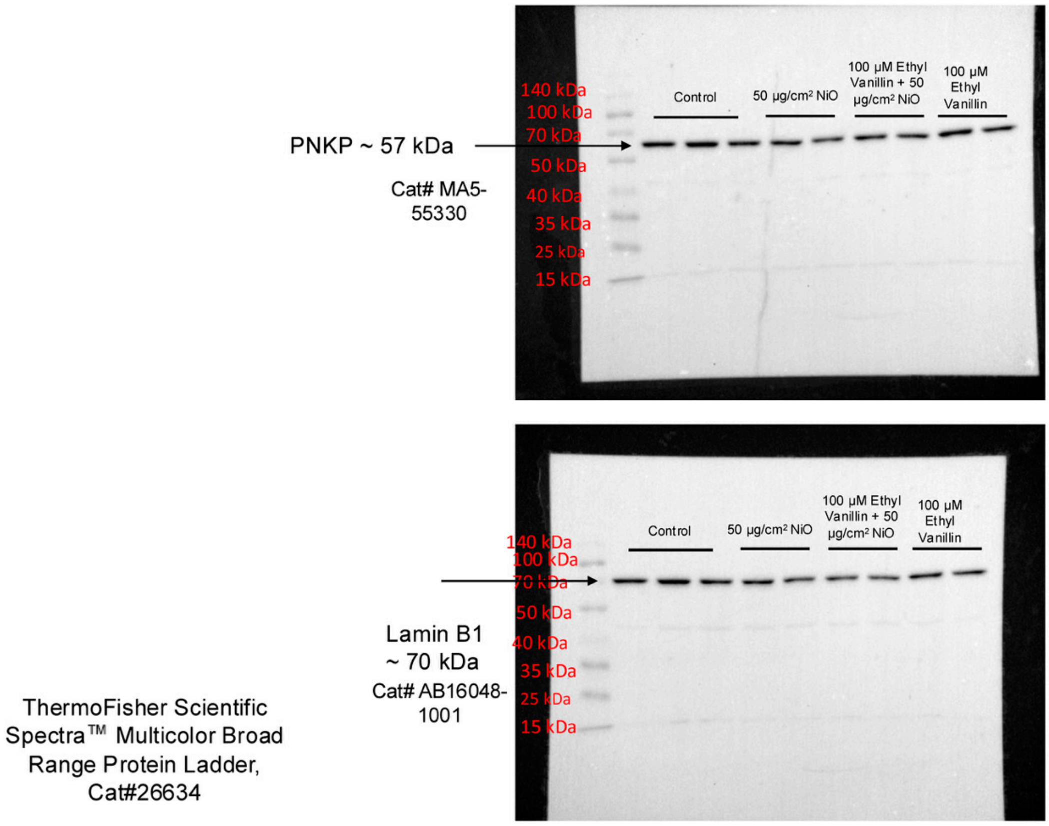

Supplementary Figure S3. Full-length, uncropped PNKP and Lamin B1 immunoblots with molecular-weight markers for samples from control, ethyl vanillin, and NiO treatments from Fig 4C.

TABLES

Vanillin Dose in µM (V)	WS23 Dose in µM (W)	Mixture Ratio	Effect (Fa)	CI Value
500	500	V:W = 1:1	0.48617	0.05093
250	750	V:W = 1:3	0.43132	0.12263
200	800	V:W = 1:4	0.43180	0.12619
100	900	V:W = 1:9	0.40630	0.18202
50	950	V:W = 1:19	0.47606	0.08363
40	960	V:W = 1:24	0.35867	0.33450
20	980	V:W = 1:49	0.38706	0.23922
750	250	V:W = 3:1	0.70892	0.00181
800	200	V:W = 4:1	0.81044	0.000275
900	100	V:W = 9:1	0.87347	0.0000381
950	50	V:W = 19:1	0.92158	0.00000446
960	40	V:W = 24:1	0.95574	0.000000576
980	20	V:W = 49:1	0.99326	0.00000000107
**Ethyl Vanillin Dose in µM (EV)**	**p-Anisaldehyde Dose in µM (A)**	**Mixture Ratio**	**Effect (Fa)**	**CI Value**
500	500	EV:A = 1:1	0.1187	16.3365
250	750	EV:A = 1:3	0.0676	101.921
200	800	EV:A = 1:4	0.1641	6.24430
100	900	EV:A = 1:9	0.1164	19.8935
50	950	EV:A = 1:19	0.0744	83.3364
40	960	EV:A = 1:24	0.0574	185.722
20	980	EV:A = 1:49	0.13	14.2022
750	250	EV:A = 3:1	0.2061	2.65026
800	200	EV:A = 4:1	0.1369	9.51028
900	100	EV:A = 9:1	0.155	6.33097
950	50	EV:A = 19:1	0.1789	4.02944
960	40	EV:A = 24:1	0.2368	1.63921
980	20	EV:A = 49:1	0.1244	11.8205

Supplementary Table S1. Exact component concentrations, ratios, fraction affected (Fa), and combination index (CI) for all binary mixtures:

Figure Panel	BEAS2B Cell Passage No	Biological Replicates *(n)*	Technical Replicates	Data Point Representation	Tukey Adjusted P Value vs. Control	Data Exclusion
1A	10	2-4	2	Percent viable cells (Mean ± SEM)	P < 0.05 at 200, 400, 600, 700, 800, 900, and 1000 µM vs. Control	None
1B	10	2-4	2	Percent viable cells (Mean ± SEM)	P < 0.05 at 50, 200, 300, 400, 500, 600, 700, 800, 900, and 1000 µM vs. Control	None
1C	10	2-4	2	Percent viable cells (Mean ± SEM)	P < 0.05 at all tested mixture ratio vs. Control	None
1D	10	-	-	CI Value < 1 across all the mixture ratios (Synergy)	-	None
1E	10	2-4	2	Total ATP (nM) (Mean ± SEM)	P < 0.05 at 25, 1000 µM Vanillin and Vanillin:WS23 =9:1 vs. Control	None
2A	20	3-12	2	Percent viable cells (Mean ± SEM)	P < 0.05 at 300, 400, 500, 600, 700, 800, 900, and 1000 µM vs. Control	None
2B	20	3-12	2	Percent viable cells (Mean ± SEM)	P < 0.05 at 200, 300, 400, 500, 600, 700, 800, 900, and	None

					1000 μM vs. Control	
2C	20	6-12	2	Percent viable cells (Mean ± SEM)	P < 0.05 at EV:A mixture ratios 1:4, 3:1,4:1, 9:1, 19:1, and 24:1 vs. Control	None
2D	20	-	-	CI Value > 1 across all the mixture ratios (Antagonism)	-	None
2E	10	2-4	2	Total ATP (nM) (Mean ± SEM)	P < 0.05 at 1000 μM Ethyl Vanillin and Ethyl Vanillin:p-Anisaldehyde =3:1, 9:1 vs. Control	None
3A	14	3	-	Percent viable cells (Mean ± SEM)	No significant (P > 0.05)	None
3B	14	3	2	IL-6 (pg/mL) (Mean ± SEM)	P < 0.05 at NiO, and Ethyl Vanillin+NiO groups vs. Control	None
3C	14	3	2	IL-8 (pg/mL) (Mean ± SEM)	P < 0.05 at NiO, and Ethyl Vanillin+NiO groups vs. Control	None
4A	14	2-3	-	H2AX / Lamin B1 Densitometric fold change relative to	No significant (P > 0.05)	None

				Control (Mean ± SEM)		
4B	14	2-3	-	NEIL2 / Lamin B1 Densitometric fold change relative to Control (Mean ± SEM)	P = 0.0518 at NiO group vs Control, and P = 0.0760 at Ethyl Vanillin+NiO group vs. Control	None
4C	14	2-3	-	PNKP / Lamin B1 Densitometric fold change relative to Control (Mean ± SEM)	P < 0.05 at Ethyl Vanillin, and Ethyl Vanillin+NiO groups vs. Control	None

Supplementary Table S2. Experimental replication and statistical details for each figure panel.
